# Comparative Examination of Capercaillie (*Tetrao urogallus* L.) Behaviour Responses and Semen Quality to Two Methods of Semen Collection

**DOI:** 10.1371/journal.pone.0138415

**Published:** 2015-09-23

**Authors:** Ewa Teresa Łukaszewicz, Artur Mikołaj Kowalczyk, Zenon Rzońca

**Affiliations:** 1 Division of Poultry Breeding, Institute of Animal Breeding, Wrocław University of Environmental and Life Sciences, Wrocław, Poland; 2 Forestry Wisła; Czarne 6; 43–460 Wisła, Poland; CNRS, FRANCE

## Abstract

Artificial insemination (AI) is very helpful in solving the reproductive and biodiversity problems observed in small, closed avian populations. The successful production of fertilized eggs using AI is dependent on the collection of good quality semen. Two methods of male sexual stimulation and semen collection from captive kept capercaillie (*Tetrao urogallus* L.), one of the most seriously endangered grouse species in Europe, are compared in this study. Ejaculates were obtained either with the use of a dummy female or by the dorso-abdominal massage method. Differences in the individual responses of the males to the two methods of semen collection as well as in their semen quality were noted. Only sperm concentration (432.4 x 10^6^ mL^-1^ with dummy female and 614.5 x 10^6^ mL^-1^ for massage method) was significantly affected by capercaillie stimulation method. Sperm motility and morphology were not affected (P≥0.05). Thus, for semen collection from captive kept capercaillie both methods can be used successfully. The dummy female can be an alternative to dorso-abdominal massage method, commonly used for semen collection from domesticated bird species.

## Introduction

In the past few decades, there has been an increasing interest in assisted animal reproduction in both domesticated and captive bird populations. The reasons and aims of it application depends mainly on species and management system. In the domesticated bird species (named poultry), the necessity of using the most common method of assisted reproduction, i.e. artificial insemination (AI) depends on many factors including: when significant weight differences exists between the male and female (commercial turkey reproductive flocks [1], [2], and more often in chicken broiler breeders [3]); when the breeder flock is kept in cages [4]; to create the intergeneric hybrids [5], [6], [7]; to detect and eliminate some diseases and pathogens transferred during natural mating, particularly in waterfowl possessing intromittent copulatory organs [8], (also named spiny penis [9]); and to eliminate injury of aged, infirm, or behaviourally incompetent males.

The assisted reproduction techniques have become more popular for wild, nondomestic bird species [10], especially those at risk of extinction, kept in zoo gardens, or in closed breeding centres [11], particularly taking into consideration a new mission of captive breeding in gene pole preservation *ex situ in vivo*. Witzenberger and Hochkirch [12] noted that most zoos contribute to animal conservation, scientific research, and public education. Many zoos participate in special breeding programs within the “European Endangered Species Programmes”. In small, closed populations reproductive problems are associated with inbreeding, incompatible pairings, or mating preference. In such cases the AI, recognized as the least stressful and invasive method of assisted reproduction [13], become a very useful tool. Through AI, there is an improvement in the fertility rates and number of offspring obtained from particular individuals, genetic exchange between populations without transferring the live animals, and the establishment of gene banks in the form of frozen semen [14], [15]. When AI is practiced, one of the most important traits determining further fertility success is the quality of the collected semen, which depends, among other, on semen collection procedure.

Although according to Immler and Birkhead [16], avian semen samples are difficult to collect, largely because the majority of bird males do not possess a phallus, several different semen collection procedures have been described for the various species. The most common is the dorso-abdominal massage method elaborated by Burrows and Quinn [17], formerly for turkeys and chicken, but later on modified, adopted and widely used for other domesticated [18], [19] and wild bird species [20], [21]. Successful semen collection using this method requires the proper male management and experienced and skilful semen collectors, who manually evoke phallic tumescence and ejaculation.

Other methods, recognized as the less stressful and welfare friendly to birds, are these that evoke natural male sexual behaviour. Differences exist in what provokes male courtship behaviour (tooting), mating attempts, and ejaculation. Semen collection by male stimulation by a female’s presence has been already used for Japanese quail [22], Muscovy duck [23], emu and ostrich [24]. For the last two species, several methods of male sexual stimulation were described: by dummy (named also artificial vagina or artificial cloaca method) [25, 26], teaser female [27], and non-teaser (human) method (male displayed courtship behaviour directed towards human [28], [29].

For semen collection from Muscovy drake [30], [31], as well as from nondomestic bird [32], the electro-ejaculation method has been applied. However, this procedure causes considerable stress to male, since he has to be anesthetized or physically restrained by human, therefore this method of semen collection is practiced rather sporadically.

Just for gross sperm morphology studies and comparison between species Immler and Birkhead [16] described a “non-invasive method” of semen collection from the wet part of the fresh faeces, while Lüpold et al. [33] collected semen from the distal end of seminal glomera (at the end of deferent ducts) of birds dissected for other experimental purposes. It is obvious that both mentioned methods can be applied only for cognitive experiments but not in AI practice

In our previous experiment carried out on capercaillie we collected semen by the modified dorso-abdominal massage method [34]. In the present experiment we wanted to evaluate the efficiency of male stimulation by a dummy method, as stimulation is more similar to natural mating, and probably less stressful to capercaillie males, and to compare the effectiveness of two semen collection methods and their impact on ejaculate quality.

## Materials and Methods

### Ethics statement

The National Forestry in Wisła District got permission (DOP-OZGIZ.6401.03.171.2011. km, dated on: May 10, 2011; expiry date: December 31, 2021; issued by the General Director of Environmental Protection) for keeping, reproduction and collection of the biological materials for experimental purposes, every year up to 50 adults capercaillie (*Tetrao urogallus*) and 150 juvenile birds in the Capercaillie Breeding Centre in Wisła, Poland. Described experiment was approved by the II Local Ethics Commission for Experiments Carried on Animals (Permit: NR 31/2010). The authors assert that all procedures (including birds’ management) contributing to this work complied with the ethical standards, and that any field studies involving the endangered species were performed in this experiment.

### Males’ management

The experiment was carried out in the Capercaillie Aviary Breeding Centre at Wisła Forest District at Silesian Beskids in Southern part of Poland. Thirteen capercaillie males, between the ages of two and eleven years old were used for semen collection. Each male was housed individually in the roofed aviary box 4.0 m x 7.0 m and 3.0 m (width, length, height) and kept under natural light [35]. Fresh nourishment and water were provided daily early morning, always by the same caretakers.

### Methods of male stimulation for semen collection and their responses

Semen was collected from each male four times a week, day by day with one day interval, alternately by two methods: one day—by dorso-abdominal massage technique [34], adopted from Burrows and Quinn [17], the next succeeding day—male stimulation by dummy female. Two different dummies were constructed and tested for male stimulation. The first dummy was made from polyurethane foam painted to resemble the plumage of capercaillie female (S1 Fig). Basing on the ambivalent male behaviour toward this first dummy, a second dummy was prepared from a female carcass that was stuffed and positioned in a sexually receptive position (S2 Fig).

During semen collection by massage method the time and response (intensity of excitation demonstrated by phallus appearance and time necessary for ejaculation) were noted and scored as follows: 4 pts—quick, spontaneous reaction (intensive, immediate phallus enlargement and erection) ended with ejaculation within 10 sec; 3 pts—positive, but slower reaction, ejaculation occurred after 30 sec; 2 pts—male could be catch (while tried to attack the birds’ keeper), but expressed any positive reaction to massage; 1 pt.—male catching attempt failed. When the stuffed dummy female was used, we distinguished the following reactions: 4 pts—quick, spontaneous reaction on dummy, mating attempt almost immediately; 3 pts—interest toward dummy, reaction after 30 sec, mating attempt; 2 pts—reaction longer than 30 sec, male ascend on the dummy, but no mating attempt; 1 pt.—lack of positive reaction, any interest in dummy, sometime male expressed anxiety and fear. In the dummy method, at the moment of intense male excitation and mating attempt he was quickly taken from the dummy and immediately thereafter, the slight press of the lateral walls around cloaca initiated ejaculation. For some males, after interrupting mating attempt a short dorsal massage had to be applied to induce ejaculation. In both methods ejaculated semen was collected to 1 mL glass tubes warmed up by holding in the collector hand. All procedures were performed by the same persons and in the same rhythm, in order to avoid unnecessary stress.

### Semen evaluation

In the freshly collected individual ejaculates the following traits were examined: 1. volume (with automatic pipette); 2. sperm concentration—by haemocytometer method, using a 3% eosin-NaCl solution (v/V) and Thoma-type grids; 3. motility—with the use of Sperm Class Analyzer SCA®, Microptic, Barcelona, Spain); 4. the integrity and morphology of the spermatozoa—on the basis of histological smears, vital stained with nigrosin-eosin (n = 300 cells per slide), evaluated at 1250x under a light microscope (Nikon Eclipse E 100) and expressed as the percentage of particular forms of spermatozoa (300 cells = 100%) [35]; 5. the Semen Quality Factor (SQF) calculated according to the following pattern: sperm concentration (n x10^6^ mL^-1^) x ejaculate volume (mL) x live normal spermatozoa (%) /100% [36]. To reduce error from subjective scoring, each ejaculate was evaluated by the same, well experienced person.

### Statistical analysis

Obtained data were analysed statistically with ANOVA, while the significance of differences were verified by Duncan’s multiple range test (Statistica, version 8.0, StatSoft, Inc., Kraków, Poland, sp. z o.o.).

## Results and Discussion

Using both methods of stimulation a specific sexual behaviour of capercaillie males, allowing their catching, stimulation and semen collection, was observed. With the massage method semen could be collected only from tooting males, who during the reproductive season tried to attack the protector entering to their aviary, what enabled their catching and performing the massage procedure. Based on our observations it is hard to agree with statement of Milonoff et al. [37], that capercaillie males who show threatening behaviour toward humans or without hesitation copulate with stuffed females, are deviant. Attack attempts toward bird protectors, indeed, sometimes rather aggressive, were observed exclusively during the reproductive season. We assumed that such males actually view the bird protectors as potential competitors, and therefore tried to attack them. Males that did not express a tooting behaviour (young or dominated by other males) were skittish and usually attempted to escape from humans. Similar behaviour toward humans was also observed outside the reproductive season for the majority of our capercaillie males.

Out of 13 males designated to the experiment, good quality semen was produced by nine individuals, described and compared in this paper. In our initial attempts, when the first dummy made from polyurethane foam was used, only one young male that had any previous contact with alive capercaillie female expressed the willingness and tried to mount the dummy. Some of males experienced in natural mating approaching the dummy, watched it, but did not tried to mount on it, while the others were very frightened and attempted to escape. Therefore, in the further attempts we used a stuffed capercaillie female dummy. Also, with respect to this dummy, a different behaviour and reactions were observed: the immediate, or after 30 seconds mounting on dummy and mating attempts (83.3% responses), frightening or aggression (16.7% responses). In case of massage method 91.9% of collections ended with ejaculation within 30 sec. The remaining attempts (8.1%) failed (Table 1).

**Table 1:** Reactions of capercaillie male (Tetrao urogallus L.) depending on sexual stimulation method.

Maleno	Male age (years)	Method of stimulation	Attempts of semen collections (No)	Reactions (Number / Percent) ^1)^				Average ±SD
				4 pts	3 pts	2 pts	1 pt.	
85^●^	2	dummy	3	1/33.3	2/66.7	−	−	3.3±0.6
		massage	3	2/66.7	1/33.3	−	−	3.7±0.6
72	3	dummy	9	9/100.0	−	−	−	4.0^**a**^ ^2)^±0.0
		massage	9	9/100.0	−	−	−	4.0^**a**^±0.0
79	3	dummy	10	5/50.0	3/30.0	1/10.0	1/10.0	3.2^**b**^ ±1.0
		massage	8	5/62.5	3/37.0	−	−	3.6^**b**^±0.5
66^●^	4	dummy	1	−	−	−	1/100	1.0
		massage	1	−	−	−	1/100	1.0
67	4	dummy	8	6/75.0	2/25.0	−	−	3.8^**ab**^±0.5
		massage	8	8/100.0	−	−	−	4.0^**a**^±0.0
68^●^	4	dummy	2	−	−	1/50.0	1/50.0	1.5±0.7
		massage	2	−	−	1/50.0	1/50.0	1.5±0.7
58	5	dummy	11	5/45.5	6/54.5	−	−	3.5^**ab**^±0.5
		massage	11	11/100.0	−	−	−	4.0^**a**^±0.0
25^●^	6	dummy	4	−	−	1/25.0	3/75.0	1.3±0.5
		massage	4	3/75.0	1/25.0	−	−	3.8±0.5
49	6	dummy	13	10/76.9	2/15.4	−	1* ^3)^ /7.7	3.6^**ab**^±0.9
		massage	12	12**^3)^/100.0	−	−	−	4.0^**a**^±0.0
50	6	dummy	7	2/28.6	1/14.2	2/28.6	2/28.6	2.4^**c**^±1.3
		massage	7	2/28.6	3/42.8	2/28.6	−	3.0^**c**^±0.8
51	6	dummy	12	9/75.0	3/25.0	−	−	3.8^**ab**^±0.4
		massage	12	12/100.0	−	−	−	4.0^**a**^±0.0
16	10	dummy	10	9/90.0	1/10.0	−	−	3.9^**a**^±0.3
		massage	10	8/80.0	2/20.0	−	−	3.8^**ab**^±0.4
20	10	dummy	9	9/100.0	−	−	−	4.0^**a**^±0.0
		massage	9	7/78.8	2/22.2	−	−	3.8^**ab**^±0.5
**Total/ Average**		dummy	**99**	**65/65.7**	**20/20.2**	**5/5.0**	**9/9.1**	3.4^**b**^±1.0
		massage	**96**	**79/82.3**	**12/12.5**	**3/3.1**	**2/2.1**	3.8^**a**^±0.6

^1)^ Legend to scoring of capercaillie reaction:

–Dummy method:

4 pts—quick, spontaneous reaction on dummy female, mating attempt,

3 pts—proper interest in dummy after about 30 sec, mating attempt,

2 pts—slower reaction, longer than 30 sec, expressed by male interest in dummy female and ascending on it, but no willingness to mate,

1 pt—lack of positive reaction on dummy female.

–Dorso-abdominal massage method:

4 pts—quick, spontaneous reaction and voluntary ejaculation after,

3 pts—positive, but slower reaction after 30 sec, ejaculation by applying a delicate pressure on both sides of cloaca,

2 pts—male allowed to be catch, but lack of positive reaction and ejaculation,

1 pt—male catching failed.

^●^ low number of collected ejaculates (less than 7), insufficient ejaculate volume and/or lack of positive respond to one of the tested method enabled the comparison of these males in Table 2;

^2)^ means in columns for the same stimulation method followed by different superscripts indicate significant differences between males (P<0.05);

^3)^ male aggression: *toward dummy female; **toward bird protector attempting catching.

In the scale 1–4 of males’ respond to sexual stimulation method, the dorso-abdominal massage was significantly (P<0.05) more effective comparing to stuffed dummy female (i.e. the 2^nd^ dummy)—(3.8 vs 3.4, on average; Table 1). In the massage method we did not observed the male age effect on reaction and excitation intensity. However, individual susceptibility to stimulation method was noted: some males (#49, #51, #58, and #67) preferred massage method, the other (#20) produced ejaculates of good quality when dummy female was applied. Males #16 and #72 expressed the positive reactions regardless the way of stimulation, while males #66 and #68 were insensible to any of tested method. In both methods used and for different males, during the entire experimental period it happened that no semen was obtained despite male’s proper excitation (indicated on the basis of cloaca appearance). Observed differences in males’ reaction to semen collection method might suggest the necessity of their different treatment during the reproductive season in order to increase the efficiency and success of semen collection. The differences in male reaction and quality of collected semen depending on stimulation method were also observed for ganders [36] and ostriches [26], [27]. In the last mentioned species authors stated any differences in ejaculates collected by dummy and teaser method, but similar as in our study, the variations between individuals were considerable.

Except sperm concentration (432.4 x10^6^ mL^-1^ with dummy female and 614.5 x10^6^ mL^-1^, for massage method), the type of collection method had no significant effect on the average values of majority of sperm quality characteristics (Table 2). Regardless, we consider both methods of capercaillie semen collections acceptable for use in AI.

**Table 2:** Characteristics of capercaillie (Tetrao urogallus L.) semen depending on male stimulation method (means; ± SD).

Maleno	Male age (years)	Method of stimulation	No of samples	Semen volume (μL)	Sperm concentration (n x10^6^ mL^-l^)	Motility (%)	Morphological forms of spermatozoa (%)			SQF^1)^
							Live in total	Live normal	Deformations in total	
72	3	dummy	9	68±21	313.3±148.7	79.2±10.3	95.0±1.9	40.0±11.4	55.0±10.6	8.5±5.8
		massage	9	60±17	560.0±441.8	77.0±8.5	96.2±2.3	35.9±11.3	60.3±9.4	16.5±20.4
79	3	dummy	8	96±30	178.3±85.8	88.4±4.7	96.1±3.2	56.2±8.5	39.9±9.5	8.5±3.3
		massage	8	80±13	122.5±77.2	90.0±6.5	95.6±1.6	52.4±17.5	43.2±18.3	4.6±2.6
67	4	dummy	8	47±11	236.7^b^ ^2)^±115.6	84.8±6.4	92.8±4.3	62.2±11.5	30.7±10.3	6.7±3.2
		massage	8	36±29	633.3^a^±260.5	77.0±11.0	96.4±2.4	67.2±8.6	29.3±10.0	12.3±10.1
58	5	dummy	11	106±53	411.8±84.5	80.5±9.4	95.9±2.4	52.3±3.8	43.7±3.7	20.4±11.5
		massage	11	145±95	287.5±158.5	83.7±5.0	95.3±2.9	56.8±7.7	38.6±7.8	22.3±15.4
49	6	dummy	12	124±49	345.0.0±141.6	84.6±5.6	96.7±2.3	71.5±11.4	25.3±10.6	28.0±11.3
		massage	12	111±38	569.2±264.9	80.1±6.8	95.3±3.8	67.1±9.7	28.2±9.7	44.1±35.1
50^3)^	6	dummy	3	40±20	560.0±466.1	77.6±13.8	93.2±5.7	53.2 ±5.7	40.0±10.2	24.3±21.0
		massage	5	96±107	260.0±105.8	75.7±7.2	97.1±0.8	66.4±14.3	30.7±13.6	16.7±14.5
51	6	dummy	12	143±31	675.8±417.3	84.3±6.8	95.8±3.8	68.1±10.4	27.6±12.1	72.2±54.8
		massage	12	109±30	781.0±446.4	90.5±4.7	95.5±6.3	65.4±5.8	30.1±4.8	62.5±48.3
16	10	dummy	10	46±14	490.0±325.7	89.3±3.5	95.7±3.0	55.5±14.5	40.2±12.9	11.2±6.4
		massage	10	32±15	510.0±213.7	87.5±2.7	93.4±3.2	47.8±14.1	45.6±15.1	9.6±10.2
20	10	dummy	9	62±26	704.3^b^±341.5	82.7±8.7	94.6±2.4	58.2±14.3	36.4±16.1	28.1±23.4
		massage	9	32±15	1417.4^a^±797.9	81.0±8.0	97.2±1.9	57.6±14.6	39.5±14.3	27.8±17.7
**Total/Average**		**dummy**	**82**	**91±48**	**432.4** ^b^ **±296.9**	**84.2±8.6**	**95.4±3.0**	**58.7±14.1**	**36.8±14.0**	**25.5±31.5**
		**massage**	**84**	**80±59**	**614.5** ^a^ **±507.1**	**83.6±7.9**	**95.6±3.6**	**56.9±14.8**	**38.7±14.8**	**27.3±31.6**

^1)^ SQF—Sperm Quality Factor—sperm concentration (n x10^6^ mL^-1^) x ejaculate volume (mL) x live normal sperm (%) / 100%;

^2) a,b^ -means in columns for the same stimulation method, followed by different superscripts indicate significant differences between males (P<0.05);

^3)^ due to low number of the collected ejaculates the male #50 was not recognized in the statistical evaluations.

In goose [38], the strain and collection method also significantly affected sperm concentration, but regarding the most important trait—sperm number per ejaculate, a clear advantage of semen collection method could not be indicated. Also in our study we did not observed significant differences in respect to SQF (Sperm Quality Factor, comprising three, the most important semen characteristics) both, in values average for compared methods (25.5 for dummy vs. 27.3 for massage method) and within individual capercaillie. As we stressed in our last article related to captive kept capercaillie reproductive potency [39], the SQF indicates the number of live normal sperm in one ejaculate. Assuming that for successful AI purposes the ejaculate quality has to be at minimum 10 SQF [40], the majority of males produced sufficiently good ejaculates to be used for AI or gene pool preservation, despite stimulation method. The average SQF varied from 4.6 (the worse male #79 and massage method) to 72.2 (male #51 and dummy method; Table 2).

Basing on the results obtained, we can conclude that for semen collection from captive kept capercaillie both methods have a similar efficiency and can be used successfully however, for sample collection into gene bank system the dorso-abdominal massage technique is more advantageous due to its preciosity and simplicity. The dummy female can be an alternative to dorso-abdominal massage method, commonly used for semen collection from domesticated bird species. Moreover, having several males the method more effective and preferred by a particular individual can be applied, allowing optimize the quantitative and qualitative semen traits.

## Supporting Information

S1 Fig Capercaillie male on the polyurethane dummy. (JPG)

S2 Fig Capercaillie male on the stuffed dummy. (JPG)
