# Supplementary figures and images for: Comparative Examination of Capercaillie (Tetrao urogallus L.) Behaviour Responses and Semen Quality to Two Methods of Semen Collection
Source: PLoS One. 2015 Sep 23;10(9):e0138415. doi: 10.1371/journal.pone.0138415 (PMC4580405; doi:10.1371/journal.pone.0138415)

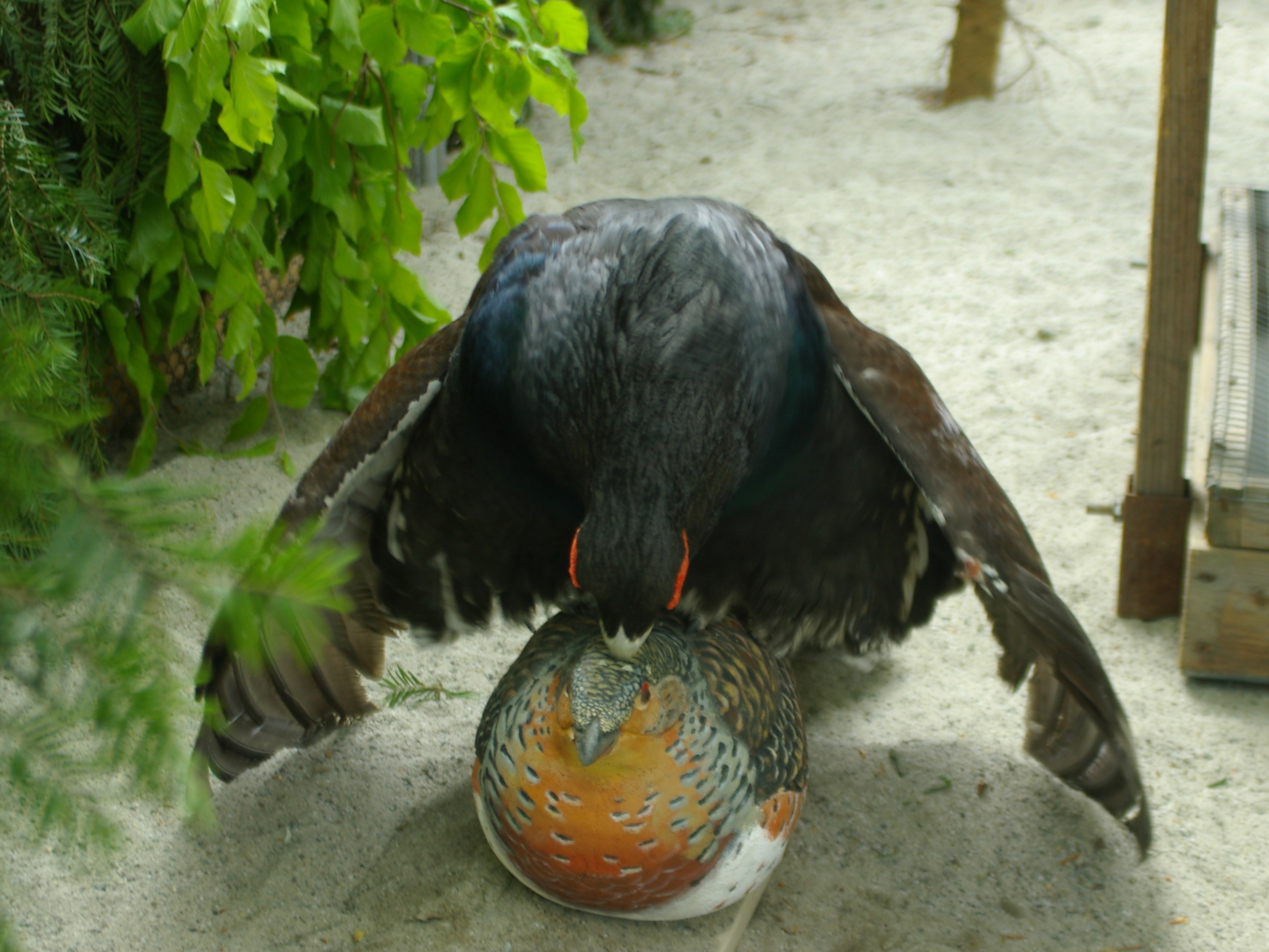

Supplement: S1 Fig — (JPG) [file pone.0138415.s001.jpg]

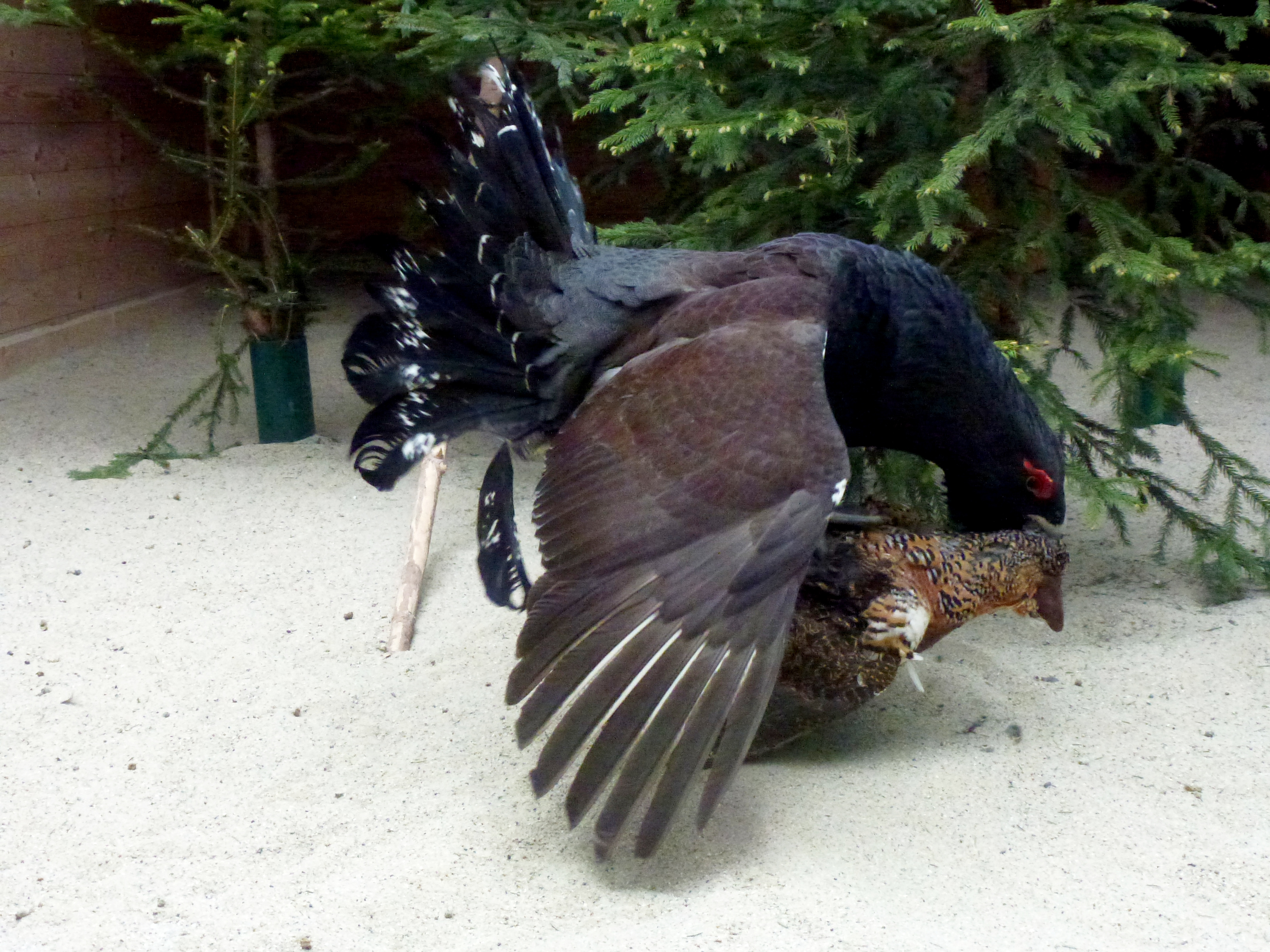

Supplement: S2 Fig — (JPG) [file pone.0138415.s002.JPG]
